# Direct Air Capture Using Aqueous Amino Acid Solvents in a Crossflow Absorber

**DOI:** 10.1021/acs.iecr.5c02551

**Published:** 2025-12-22

**Authors:** Jorge Gabitto, Abishek Kasturi, Gyoung Gug Jang, Radu Custelcean, Costas Tsouris

**Affiliations:** 1 Chemical Engineering Department, 6717 Prairie View A&M University, Prairie View 77446, United States; 2 6146 Oak Ridge National Laboratory, P.O. Box 2008 Oak Ridge, Tennessee 37831, United States

## Abstract

Carbon dioxide (CO_2_) is the most abundant of all greenhouse gases (GHGs). CO_2_ levels in the atmosphere are 50% higher than in the preindustrial era, trapping heat. CO_2_ removal from the atmosphere by direct air capture (DAC) is needed to achieve the internationally agreed global temperature goals. The most common CO_2_ capture technology is absorption by amine-based solvents in packed columns. Amino acid solutions have recently gained attention due to their advantages over traditional amine solvents. To be implemented effectively, DAC industrial processes need to handle large airflow rates in separation absorbers. The large air and solvent flow rates preclude the use of countercurrent columns due to high-pressure drops and the occurrence of flooding. Crossflow air–liquid absorbers are used to handle large air and liquid volumes due to their lower-pressure drop. The objective of this work is to study the influence of crossflow absorber geometric parameters and operating conditions on product formation and process efficiency. An already derived theoretical model for countercurrent absorbers has been modified to simulate the operation of a crossflow DAC absorber. The predictive model was implemented into a computer code that was used to study the efficiency of the processes as geometric equipment dimensions and operating parameters vary. Practical suggestions are made to design more efficient DAC processes.

## Graphical abstract

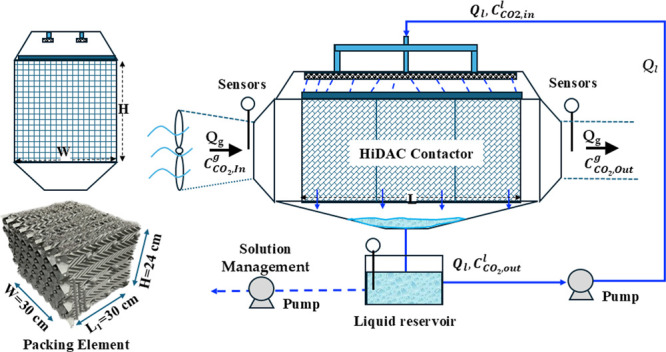

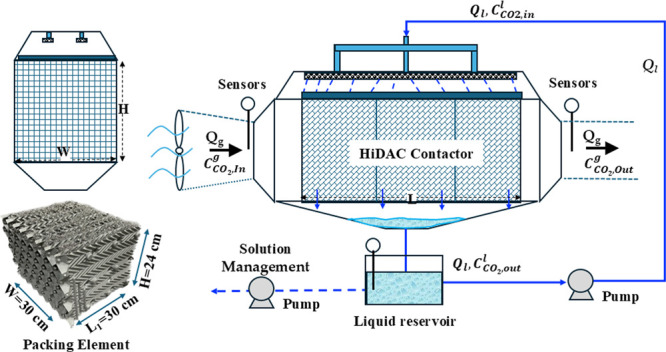

## 1 Introduction

The CO_2_ levels measured in 2023 were 50% higher than those of the preindustrial era.[Bibr ref1] 2023 was also found to be the warmest year in the 174-year observational history. This shattered the record of the previous warmest years, 2016 and 2020.^1^ Significant reductions in CO_2_ emissions are necessary to achieve the goals of keeping global temperature increase below 1.5 °C by 2030 and achieving net zero emissions by 2050.^2^ Pre- and postcombustion CO_2_ capture from large point sources can slow down the rate of increase, but only the direct removal of CO_2_ from air, or direct air capture (DAC), can lower atmospheric CO_2_ concentrations.^3–5^

The most established industrial technology for CO_2_ sequestration from concentrated sources is reactive absorption using selective solvents or solvent blends.^6–16^ Amines and various amine-based blends have been widely studied despite several known limitations associated with these solvents.^6–10^ Recently, the use of amino acid solutions, such as potassium sarcosinate, has been proposed because amino acids are not corrosive, have lower volatility, and are chemically more stable than typical amine solvents.^11–18^ Aqueous amino acid solvents can also be thermally regenerated at moderate temperatures between 100 and 120 °C, thus having the potential to utilize waste heat from power plants. Several of these reactive absorption processes are carried out in gas–liquid packed-bed columns that work in different flow arrangements. The gas and liquid phases flow in cocurrent, countercurrent, or crossflow pathways. Packed columns allow operation using new structure packings with very high surface area and void fractions.^19–21^ Countercurrent packed columns are typically used for gas–liquid separations due to high heat and mass transfer rates, high mass transfer area, and a relatively constant concentration gradient throughout the tower.^22,23^ Due to its comparative advantages, there is a rich literature about countercurrent packing separation towers compared to other configurations including cocurrent, crossflow, radial, and hybrid arrangements.^24–27^ However, countercurrent operation is not the most efficient arrangement for DAC applications that involve large flow rates of gas and liquid streams. Crossflow operation, where the liquid phase flows from top to bottom by gravity while the gas phase enters at right angles from the side of the column, is more economical for large gas and liquid flow rates because it causes less pressure drop, and is less affected by flooding, or flow reversal, which is a significant problem in counterflow operation.^24^ These conditions make crossflow configurations attractive for large operations that deal with low CO_2_ concentration feeds. Recently, DAC absorption by crossflow operation has been proposed by several authors.^19–21^ However, the literature for crossflow is not as well-developed as the literature for counterflow applications.^22,23^

There are only a handful of references related to DAC applications of crossflow contactors.^19–21^ Due to the relatively low concentration of CO_2_ in air, a large flow rate of air needs to be processed in DAC operations. For the solvent to be saturated in one pass through the contactor, its residence time needs to be sufficiently high, requiring a contactor of over 20 m height.^28,29^ In this work, the height of the contactor used in the experiments is much smaller than 20 m; thus, the solvent is continuously recycled through the contactor until it is saturated. CO_2_-saturated solvent is then regenerated and reused.^19–21^

The objective of this work is to develop a tool that can be used to determine optimal conditions for CO_2_ absorption from air using an amino acid solution in crossflow absorption operations. We compare experimental data obtained at ORNL and simulation results calculated by a 2D model developed by the authors.^17^ This work has been structured in two steps. First, an existing experimental absorber has been used to validate a theoretical simulation model. Second, after establishing that the model is adequate to describe the influence of the different operational and geometric process parameters, we used the model to predict the process performance under various conditions.

The following strategy was used in this study: we identified a minimum set of parameters that affect the absorber performance. We selected five parameters: gas and liquid phase superficial velocities (*u*_g_ and *u*_l_), height (*H*) and length (*L*), and temperature (*T*). All these parameters can be manipulated as input model parameters. The selection of superficial velocities to account for the flow of the gas and liquid phases deserves explanation as routinely flow rates are used instead. The influence of individual variables is studied by varying only one of them at a time, while keeping the others constant. The gas and liquid flow rates are related to the geometric parameters of the absorber through the fluid flow areas; therefore, we considered the superficial velocity, which is not affected by the geometric parameters, as a better indication of the hydrodynamic state of the absorber compared to the flow rates.

## 2 Materials and Methods

### 2.1 Experimental Part

Model validation was done using experimental data measured at a crossflow absorber located at the Oak Ridge National Laboratory (ORNL).^17^ We include a brief description of the experimental setup and procedures.

Potassium hydroxide pellets and sarcosine (both 99% purity) were purchased from Sigma-Aldrich and used to prepare the potassium sarcosinate (K-SAR) solutions utilized. A prototype air/liquid contactor, referred to as the high flux direct air capture (HiDAC) contactor, was constructed using polyvinyl chloride Brentwood structured packing (Model: XF75 Pro). Detailed descriptions of the contactors used in this study, including their geometries, are available in the literature.^17^ The specific surface area of the HiDAC absorber is 900 m^2^/m^3^ and has a void fraction of 93.7%.

A wind tunnel setup was constructed at ORNL to house the gas–liquid contactor and conduct CO_2_ loading experiments. An illustration of this setup is provided in Figure 1. The crossflow wind tunnel, measuring 1.30 m × 0.34 m × 0.34 m, was used to evaluate CO_2_ uptake (GMP343 sensors, Vaisala) and to study the effects of air velocity (Dwyer 641RM Air Velocity Probe), air temperature, solvent pH (transmitter: Sensorex TX100; probes: SG200CD), humidity uptake (HX94CW, Omega), and pressure drop (PX459-005DWUI, Omega) across the contactor. Air enters the wind tunnel horizontally, while the solvent is introduced perpendicularly, flowing from the top to the bottom of the HiDAC absorber, which is positioned at the tunnel’s center. CO_2_, humidity, and air velocity sensors, along with thermocouples, were installed at both the inlet and outlet to monitor CO_2_ concentration, humidity uptake, air velocity, and temperature. The solvent was collected in the bottom reservoir, sent to a tank, and continuously recirculated using a feed pump. Solvent pH was monitored in real time using a pH probe. All the solvent was continuously circulated.

**Figure 1:**
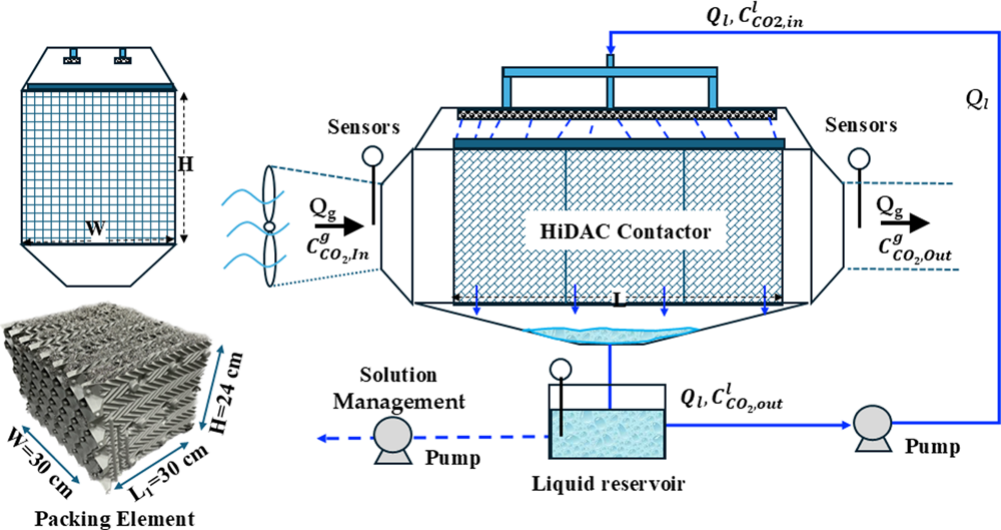
Schematic of the crossflow direct air capture wind tunnel and the HiDAC absorber.

An air blower (Turbo Blower, FDC-005A-7W, Fuji Electronic Corp. of America) used to deliver air at flow rates up to 254.3 Cubic Feet per Minute (CFM) was set up in our laboratories. The inorganic carbon content of solvent samples was measured using a total inorganic carbon analyzer comprising a CM5330 Acidification Module and a CM5017 CO_2_ Coulometer Module. The pH of the solvent in the reservoir was monitored using a pH probe. A schematic of the HiDAC absorber used in this work is depicted in Figure 1. The HiDAC absorber comprises three packing elements assembled in a horizontal arrangement (see Figure 1). The characteristics of the crossflow absorber are listed in Table 1.

**Table 1:** Packing Element Parameter Values^17^

parameter	units	values
specific surface area (*a*_t_)	m^2^/m^3^	900
void fraction (ε)	dimensionless	0.937
vertical dimension (H)	m	0.24
horizontal dimension (L)	m	0.305
width (W)	m	0.305
constant for gas phase mass transfer^9^ (*C*_g_)	dimensionless	0.293
packing constant for liquid hold-up, eq 11 (*C*_h_)	dimensionless	0.957
liquid phase mass transfer, eq 9 (*C*_l_)	dimensionless	0.971

### 2.2 Absorber Simulation Model

#### 2.2.1 Reaction Scheme

The crossflow absorber simulated in this work is depicted in Figure 1. The complex chemical reactions for CO_2_ absorption were simulated using the reaction scheme proposed by Kasturi et al.^30,31^ The main CO_2_ absorption reaction by alkaline sarcosine (SAR^–^) is given by eq 1. The reaction follows a nonelementary mechanism that results in the formation of sarcosine carbamate (SAR^–^COO^–^) and neutral sarcosine (SAR).^15,30–33^

$${CO}_{2}\left(\mathrm{aq.}\right)+2{SAR}^{-}\overset{K_{1}}{\Leftrightarrow }{SAR}^{-}{COO}^{-}+SAR$$ 1

The CO_2_ absorption reaction in alkaline solutions is given by

$${CO}_{2}\left(\mathrm{aq.}\right)+{OH}^{-}\overset{K_{2}}{\Leftrightarrow }{{HCO}_{3}}^{-}$$ 2

For high amino acid concentrations, eq 2 is negligible compared to eq 1. However, this reaction becomes important at low amino acid concentrations.^31^

At high carbamate concentrations, the carbamate (SAR^–^COO^–^) is hydrolyzed to produce bicarbonate $\left({{\mathrm{HCO}}_{3}}^{-}\right)$ and regenerate alkaline sarcosine (SAR^–^).^31^

$${SAR}^{-}{COO}^{-}+H_{2}O\overset{K_{5}}{\Leftrightarrow }{\mathrm{SAR}}^{-}+{{HCO}_{3}}^{-}$$ 3

The complete reaction scheme is described by Kasturi et al.^30,31^

#### 2.2.2 Mass and Heat Transfer Balances

This work follows the study reported by Gabitto and Tsouris.^9^ The authors developed a dynamic model to reproduce experimental findings in CO_2_ absorption by solvents under countercurrent operation without recirculation. The model comprises both mass and energy balances for the typical chemical species present in gas–liquid absorption. The liquid phase flows from top to bottom while the gas phase flows horizontally from the left to the right side of the absorber, perpendicular to the liquid flow, as shown in Figure 1. Gabitto and Tsouris developed a one-dimensional model for a countercurrent packing column. In this work, we developed a two-dimensional model for a crossflow absorber.^9^ A model summary is included here.

CO_2_ gas–liquid reactive mass transfer was modeled using the two-film theory, which assumes that mass transfer across the interface occurs solely by molecular diffusion through two thin boundary layers, one in the gas phase and one in the liquid phase.^34^ Mass transfer coefficients are defined for each phase: *k*_g_ for the gas phase and *k*_l_ for the liquid phase. The effect of chemical reaction on mass transfer is captured by an enhancement factor (*E*), which is defined as the ratio of the mass transfer rate with reaction to that without reaction.^22,23^ Chemical reactions are assumed to occur within a thin film on the liquid side of the interface.

Gabitto et al. proposed the following mass balance equations in the liquid phase.^8,9,29^

$$\varepsilon_{l}\frac{\partial C_{i}^{l}}{\partial t}=u_{l}\frac{\partial C_{i}^{l}}{\partial z}+D_{sl}\frac{\partial^{2}C_{i}^{l}}{\partial x^{2}}+k_{CO2}^{l}Ea_{w}\left(C_{i}^{l,*}-C_{i}^{l}\right)+R_{gen,i}\varepsilon_{l}$$ 4

Here, *C*_*i*_^l^ is the liquid concentration, *C*_*i*_^l,*^ is the liquid phase equilibrium concentration at the gas–liquid interphase, ε_l_ is the liquid volume fraction (*V*_l_/*V*_t_), *a*_w_ is the interphase area per unit volume, *R*_gen,*i*_ represents moles of species *i* generated/consumed by interphase reaction per unit volume, *k*_CO2_^l^ is the mass transfer coefficient, *D*_sl_ is the liquid-side dispersion coefficient, and *E* is the enhancement factor.^23^

In the gas phase, a mass balance for species *i* leads to^8,9,35^

$$\varepsilon_{g}\frac{\partial C_{i}^{g}}{\partial t}=\frac{u_{g}}{L}\left(C_{i,In}^{g}-C_{i,Out}^{g}\right)+D_{sg}\frac{\partial^{2}C_{i}^{g}}{\partial z^{2}}-k_{CO2}^{l}a_{w}\left(C_{i}^{l,*}-C_{i}^{l}\right)$$ 5

Here, *L* is the absorber length, *u*_g_ is the gas superficial velocity, ε_g_ is the gas phase volume fraction (*V*_g_/*V*_t_), *D*_sg_ is the perpendicular gas dispersion coefficient, and *C*_*i*,In_^g^, *C*_*i*,Out_^g^ are the input and output gas phase concentrations, respectively.

#### 2.2.3 Chemical Reactor Energy Balance Model

The CO_2_ absorption reactions described in the reaction scheme are highly exothermic, necessitating the solution of an energy balance to account for temperature variations. Additionally, there is considerable mass transfer of water into the gas phase, which involves significant energy changes due to the latent heat required for phase change of liquid molecules.^8,9,35^ Gabitto et al. presented the energy balances for both the liquid and gas phases as follows:^35^

$$\frac{\partial T^{l}}{\partial t}=-\underset{i}{\sum }N_{i,diff}\frac{\Delta H_{vap,i}}{\underset{i}{\sum }C_{i}^{l}C_{pi}^{l}}-N_{CO2,diff}\frac{\Delta H_{R}}{\underset{i}{\sum }C_{i}^{l}C_{pi}^{l}}-U_{T}a_{w}\frac{\left(T^{l}-T^{g}\right)}{\underset{i}{\sum }C_{i}^{l}C_{pi}^{l}}$$ 6

$$\frac{\partial T^{g}}{\partial t}=\left\langle T_{o}^{g}\frac{\underset{i}{\sum }C_{i,}^{go}C_{pi}^{go}}{\underset{i}{\sum }C_{i}^{g}C_{pi}^{g}}-T^{g}\right\rangle +U_{T}a_{w}\frac{\left(T^{l}-T^{g}\right)}{\underset{i}{\sum }C_{i}^{g}C_{pi}^{g}}$$ 7

Here, *C*_pi_^g^ and *C*_pi_^l^ represent the heat capacities in the gas and liquid mixtures for the *i*th component, respectively; *C*_*i*,_^go^ is the inlet gas phase concentration of the *i*th component; *C*_pi_^go^ is the heat capacity of the *i*th component in the gas inlet stream. *U*_T_ is the global heat transfer coefficient, Δ*H*_vap,*i*_ is the heat of vaporization for the *i*th species, *T*_o_^g^ is the inlet gas stream temperature, Δ*H*_R_ corresponds to the heat released by the reaction, and *u*_g_ and *u*_l_ are the superficial velocities of the gas and liquid phases, respectively. The molar flow of component *i* due to diffusion, *N*_*i*,diff_, is calculated using the equation proposed by Gabitto et al.^9^ The molar flow of CO_2_ due to diffusion, *N*_CO2,diff_, is given by^31^

$$N_{CO2,diff}=-k_{CO2}^{l}Ea_{w}H^{cc}C_{CO2}^{g}$$ 8

Here, *k*_CO2_^l^ is the liquid-side CO_2_ mass transfer coefficient. The following assumptions are used for eq 8. The liquid phase concentration (*C*_CO2_^l^) is negligible compared to the gas phase concentration (*C*_CO2_^g^), and the resistance to mass transfer is located mostly in the liquid thin layer.

#### 2.2.4 Parameter Estimation

Implementing the crossflow packed model involves evaluating several key parameters. For completeness, a selection of these parameters is listed here; a full list is available in the referenced literature.^9,30,31,34,35^

$$k_{l}^{i}=C_{l}{\left(\rho_{l}g/\mu_{l}\right)}^{1/6}{\left(a_{t}D_{l}^{i}/4\varepsilon \right)}^{1/2}{\left(u_{l}/a_{t}\right)}^{1/3}$$ 9

Here, *k*_l_^*i*^ are the mass transfer coefficients for all chemical species in the liquid phase; *C*_l_ is the packing constant for the liquid phase; *a*_t_ represents the bed-specific area; ε is the void fraction; *u*_l_ is the superficial velocity of the liquid phase; *D*_l_^*i*^ denotes the diffusivity of component *i* in the liquid phase; and μ_l_ is the dynamic viscosity of the liquid phase. Equation 9 was adapted from Greer.^8^

A very important parameter is the liquid hold-up per unit volume (*h*_t_) given by^36^

$$h_{t}={\left(\frac{12a_{t}^{2}\mu_{l}}{\left(g\rho_{l}\right)}\right)}^{3/4}{\left(\frac{a_{w}}{\left(a_{t}\right)}\right)}^{2/3}$$ 10

where *a*_w_ is the specific wet surface area for mass transfer calculated by^8,36^

aw(at)=Ch(ρlul(μlat))0.15(atul2g)0.1whenRe⁡l<5 11

aw(at)=0.85Ch(ρlul(μlat))0.25(atul2g)0.1whenRe⁡l>5 12

Here, *C*_h_ is the packing constant for liquid hold-up and *Re*_l_ is the liquid phase Reynolds number, Rel=(ρlul(μlat)) .

The *C*_*i*_ constants that appear in eqs 9 to 12 are either provided by the packing manufacturer or experimentally determined. The custom-made structure packing elements used in this work have a high surface area (on the order of 900 m^2^/m^3^) and void fraction (∼0.937). The pressure drop in all the experiments was approximately 100 Pa/m.^17^ A complete set of parameters used in the simulations can be found in references 8, 9, and 36 and in Tables 1 and 2.^8,9,36^

**Table 2:** Crossflow Absorber Parameters

parameter	units	basic set value	range
absorber height (*H*)	m	1.0	0.305–10
absorber length (*L*)	m	1.0	0.305–3
absorber width (*W*)	m	0.305	0.1–0.5
reservoir volume (*V*_T_)	m^3^	0.04	0.01–0.20
gas superficial velocity (*u*_g_)	m/s	0.5	0.1–1.1
liquid superficial velocity (*u*_l_)	m/s	1.2 × 10^–3^	6.E-4–7.2 × 10^–3^
input CO_2_ molar fraction (*x*_CO2_)		6.E-4	3.3 × 10^–4^–1.2 × 10^–3^
initial pH		11.7	11.2–12.4
temperature	K	303	288–313
pressure	Pa	1.1E5	1.1E5–1.3E5
initial amino acid concentration	mol/m^3^	1000	1000–3000

### 2.3 Model Implementation and Numerical Solution

The crossflow absorber model was implemented by modifying a custom-made computer software developed by Gabitto et al. for dynamic simulation of a countercurrent flow absorber.^9^ FORTRAN-77 was employed for model implementation and programming. The original computer code was used as a framework for the software developed in this work. 2D discretization of the domain was implemented to numerically solve a combination of hyperbolic partial differential equations (PDEs) and nonlinear algebraic equations. A set of mass balances in the gas and liquid phases for all chemical species participating in the process (eqs 4 and 5), was solved using finite differences methods for the PDEs plus the two-phase temperatures (eqs 6 and 7), coupled with several routines for calculating kinetic parameters, mass transfer coefficients (eq 9), physical properties, hydrodynamic conditions, packed-bed properties such as hold-up, wet area, etc. (eqs 10–12). A complete description of program structure appears in ref 9. Mass balances for all liquid phase chemical species leaving the absorber into the reservoir and the pump tank were carried out to determine the concentration of those chemical species entering at the top of the absorber. The results used to study the influence of the different parameters were obtained by calculating average concentrations leaving the absorber at the bottom (solvent phase) and the side (gas phase). For scaleup purposes, the model needs to be further developed and validated for larger-scale operations to make sure that flow nonidealities, such as channeling and liquid maldistribution, are accounted for.

## 3 Results and Discussion

A custom-made computer code developed by Gabitto et al. for dynamic simulation of a countercurrent absorber was modified to implement the 2D crossflow absorption model.^9^ This model was validated through comparison with experimental data from the ORNL crossflow absorber^19^ as well as data reported in the literature.^8,17,37^

Figure 2a and Figure 2b illustrate the comparison of simulation results and experimental data, respectively. The efficiency of the absorption process can be measured in two ways. The absorption efficiency for the gas phase can be measured as the CO_2_ absorption ratio, defined as $\left(\frac{C_{\mathrm{CO}2,\mathrm{In}}^{g}-C_{\mathrm{CO}2,\mathrm{Out}}^{g}}{C_{\mathrm{CO}2,\mathrm{In}}^{g}}\right)$ . The absorption efficiency of the liquid phase is reported using the effective CO_2_ load defined as the summation of the concentrations of all compounds containing CO_2_ including ${\mathrm{SAR}}^{-}{\mathrm{COO}}^{-},{{\mathrm{HCO}}_{3}}^{-},{{\mathrm{CO}}_{3}}^{2-},\mathrm{and}{\mathrm{CO}}_{2}(\mathrm{aq.})$ divided by the initial amino acid concentration. Both ways of measuring the efficiency of the process are related as the amount of CO_2_ absorbed from the gas phase is continuously accumulated in the liquid phase.

**Figure 2:**
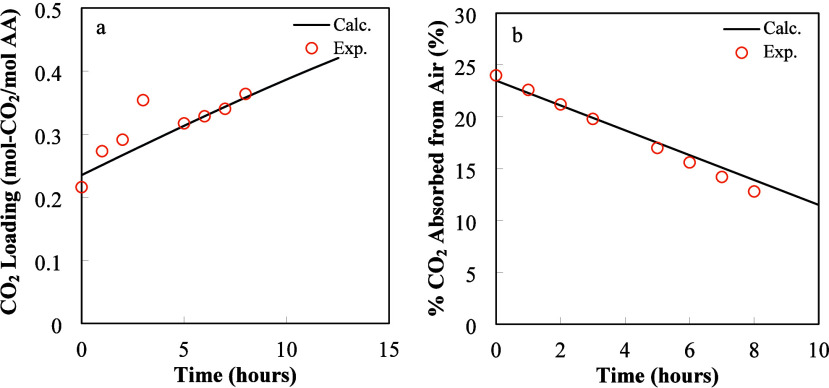
Comparisons of the time variation of CO_2_ load in liquid phase (a) and percentage of absorbed CO_2_ from the gas phase (b). Input data: *L* = 0.24 m, *W* = 0.305 m, *L* = 0.915 m, *Q*_l_ = 12 L/min, *Q*_g_ = 4831.2 L/min, and *T* = 295 K.

CO_2_ loading experiments were conducted using 1 M K-SAR in the HiDAC contactor with three packing elements (0.915 m total length, 0.24 m height, and 0.305 m width) at room temperature, with an air velocity of 1.1 m/s through the contactor and 12 LPM of solvent flow rate. The percentage of CO_2_ absorbed is defined as the CO_2_ absorption fraction multiplied by 100.

There is good agreement between calculated results and experimental data. The calculated results in Figure 2a show a continuous increase in CO_2_ load as time increases. It is shown in Figure 2b that, as time increases, the amount of CO_2_ absorbed from the gas phase decreases as the concentration of free amino acid (SAR^–^) decreases with time. Inspection of Figure 2b shows that the simulated results slightly overpredict CO_2_ absorption at long times.

A comparison between experimental data and simulation results for gas superficial velocities (*u*_g_) and temperature is shown in Figure 3. The concentration of amino acid is 3 M in all cases, and the experiments and simulations have been carried out at two temperatures, 308.1 and 268.1 K. The gas absorbed fraction decreases as the gas superficial velocity increases for the experimental data and simulation results. Also, the gas absorbed fraction increases as the temperature increases. The qualitative behavior is similar for both experimental data and simulation results. The agreement between both sets of data supports the modeling approach used in this study. However, the values of the simulation results are higher than the experimental data at high temperatures while they are lower than the equivalent data at low temperatures. This behavior may be due to errors in the estimation of mass transfer and reaction parameters vs temperature or the estimation of packing parameters, which were obtained through correlations developed for other packing geometries. The dimensions used in the simulation were the same values as in the HiDAC absorber, *H* = 0.24 m, *W* = 0.305 m, and *L* = 0.915 m. In Figure 3, the gas flow rate (*Q*_g_) was varied from 1537.2 to 5006.9 L/min, while the liquid flow rate (*Q*_l_) value was 20 L/min.

**Figure 3:**
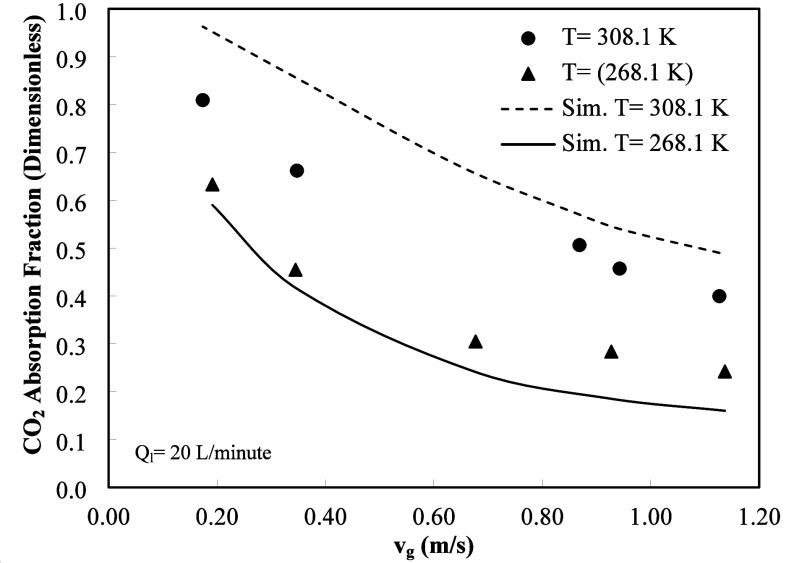
Comparison between simulation results and experimental data for temperatures 308.1 and 268.1 K vs gas superficial velocity. Absorber dimensions: *L* = 0.24 m, *W* = 0.305 m, and *L* = 0.915 m.

In this work, we started our simulations from an initial condition that proved to reduce the absorption time in similar experiments carried out in our laboratories.^17^ We first allowed liquid flow only for approximately three times the liquid phase residence time. At that time, we started the gas flow rate. The idea was to reach approximately constant liquid distribution and wetting of the solid packing before introducing the gas phase. The input of the gas phase when there was adequate wet surface area reduced significantly the time to reach hydrodynamic steady-state conditions inside the absorber. Furthermore, the introduction of the gas phase into the absorber when the wet packing area (*a*_w_) was close to the steady-state value significantly increased the mass transfer rate that is directly proportional to the value of the wet surface area.

The time variation of all chemical compounds that participate in eqs 1–3 is shown in Figure 4a,b. The concentrations of the OH^–^ and H^+^ ions are not shown as their values are very small. In Figure 4a, it is shown that the CO_2_ absorption by the alkaline sarcosine (SAR^–^) (eq. 1) was the main reaction until about 2000 s, pseudo-first order regime.^7–13^ This reaction consumed SAR^–^ while producing approximately equal amounts of carbamate (SAR^–^COO^–^) and protonated sarcosine (SAR). At longer times, the carbamate (SAR^–^COO^–^) concentration decreased by transformation into bicarbonate ions (eq 3). At times longer than 6000 s, equilibrium was reached. The total concentration of CO_2_ containing chemical compounds remained always equal to the protonated sarcosine concentration due to one-to-one transformation of the carbamate into bicarbonate (eq 3). However, the stoichiometric ratios in eq 1 for both protonated sarcosine and total CO_2_ concentration should be equal to 0.5 of the consumed alkaline sarcosine (SAR^–^). These values are about 0.6 in Figure 4a. The extra amount produced occurs because eq 3 also produces alkaline sarcosine. In this way, some sarcosine salt molecules were regenerated and participated more than once in absorbing CO_2_ molecules from the gas phase. Figure 4b shows that the fraction of CO_2_ absorbed from the gas phase decreased continuously as the liquid phase amino acid concentration decreased with time. There is a linear variation of absorbed CO_2_ fraction up to 2000 s. At longer times, the fraction decreased much faster until it became very small at equilibrium. The time variation of the different chemical species concentrations agrees well with literature results for several solvents.^8,37^ This finding confirms that the results produced by the 2D model developed in this work agree well with the literature data.^8,37^

**Figure 4:**
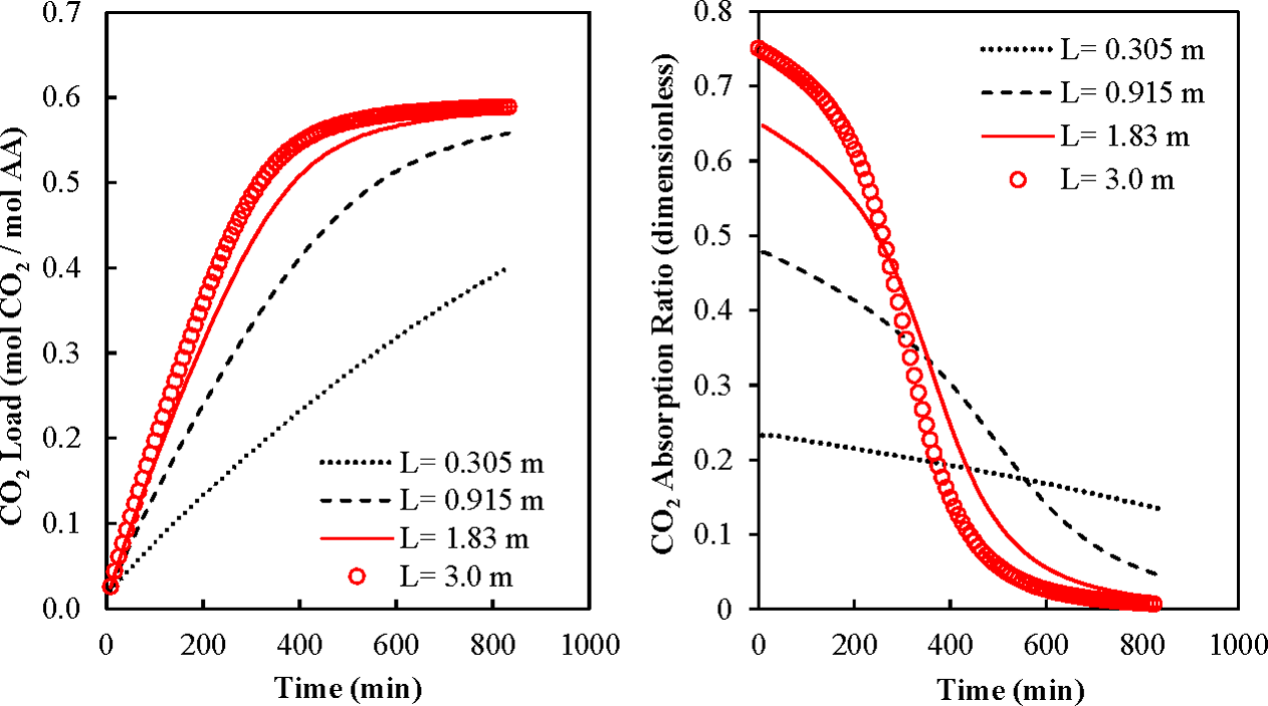
(a) Time variation of relevant chemical species concentrations in the liquid phase. (b) Time variation of the CO_2_ gas phase absorbed fraction. Input data: *H* = 1 m, *L* = 3 m, *W* = 0.305 m, *u*_l_ = 1.2 × 10^–3^ m/s, *u*_g_ = 0.5 m/s, and *C*_CO2,In_^g^ = 2.7 × 10^–5^ mol/L.

In all simulations carried out in this work, energy balances on both fluid phases allowed calculation of the corresponding temperature profiles. The local temperature values were used to calculate all temperature-dependent variables. For equal input temperatures of both fluid phases, however, the highest temperature difference observed experimentally was never more than 2 °C, and the process reached steady-state temperature values at short times. These results suggest that the heat generated by CO_2_ absorption is mostly used to evaporate the liquid solvent (water), as these are the two most important heat generating processes. The results also suggest that there was very good heat transfer between both fluid phases due to the high interfacial heat transfer area.

In Figures 5, 6, 7, and 8, we present results calculated by varying geometrical and operating parameters of the process. In all Figures, we present results showing the time variation of the CO_2_ load in the liquid phase and the CO_2_ absorption fraction in the gas phase.

**Figure 5:**
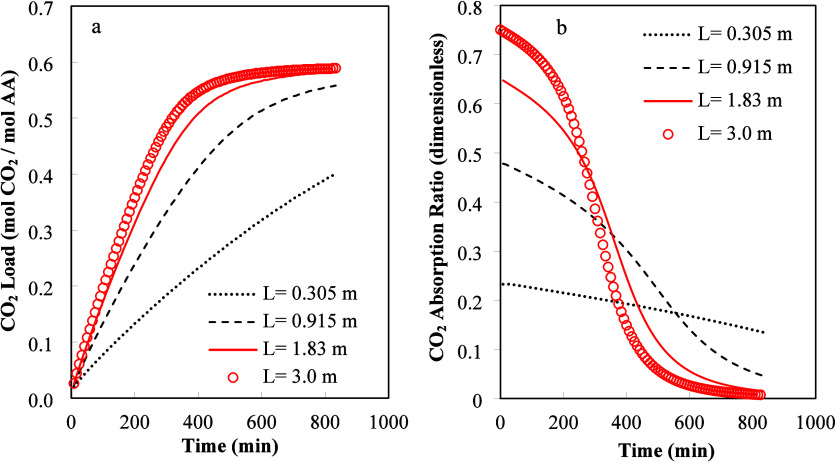
Influence of absorber length on (a) CO_2_ load in liquid phase and (b) gas absorption fraction. Input data: *H* = 1 m, *W* = 0.305 m, *u*_l_ = 1.2 × 10^–3^ m/s, *u*_g_ = 0.5 m/s, and *C*_CO2,In_^g^ = 2.7 × 10^–5^ mol/L.

**Figure 6:**
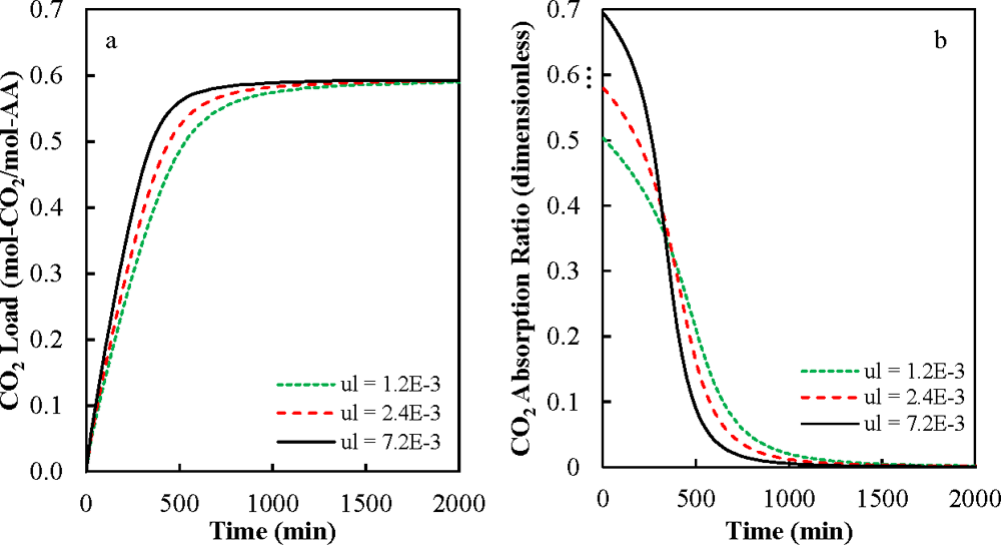
Influence of absorber height on (a) CO_2_ load in liquid phase and (b) gas absorption fraction. Input data: *L* = 1 m, *W* = 0.305 m, *u*_l_ = 1.2 × 10^–3^ m/s, *u*_g_ = 0.5 m/s, and *C*_CO2,In_^g^ = 2.7 × 10^–5^ mol/L.

**Figure 7:**
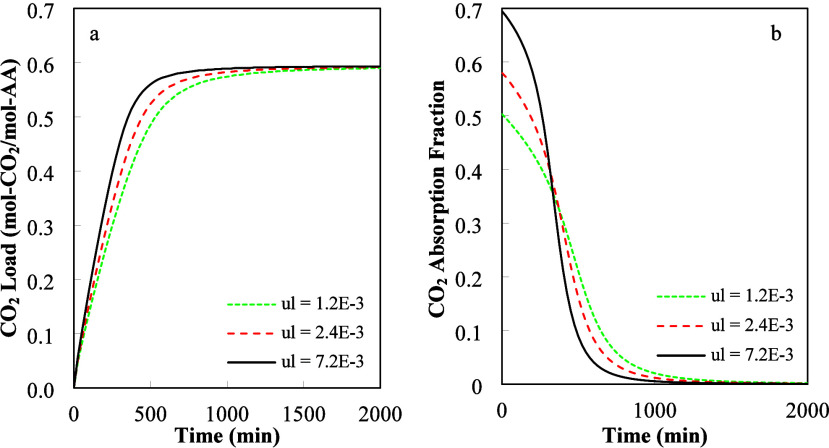
Influence of liquid superficial velocity on (a) CO_2_ load in the liquid phase and (b) gas absorption factor. Input data: *H* = 1 m, *L* = 1 m, *W* = 0.305 m, *u*_g_ = 0.5 m/s, and *C*_CO2,In_^g^ = 2.7.0 × 10^–5^ mol/L.

**Figure 8:**
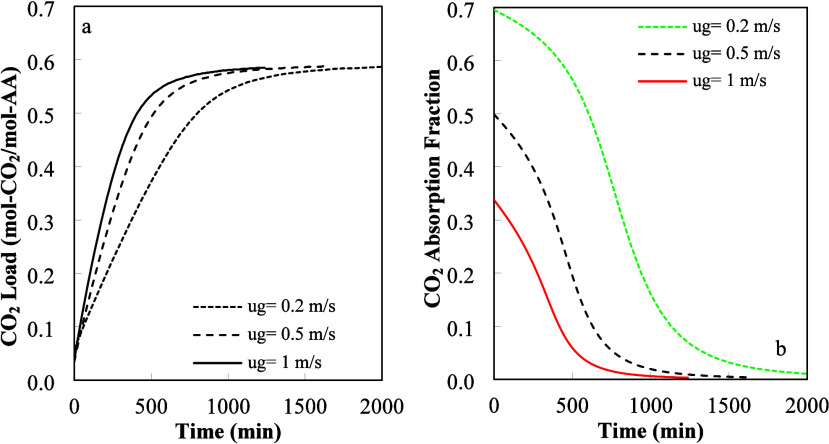
Influence of gas superficial velocity on (a) CO_2_ load in the liquid phase and (b) gas absorption fraction. Input data: *H* = 1 m, *L* = 1 m, *W* = 0.305 m, *u*_l_ = 1.2 × 10^–3^ m/s, and *C*_CO2,In_^g^ = 2.7 × 10^–5^ mol/L.

In Figure 5a,b, we varied the horizontal dimension (length) of the absorber keeping constant the liquid superficial velocity (*u*_l_) and the gas flow rate (*Q*_g_). The liquid superficial velocity was kept constant to maintain the wet surface area constant for the simulation set (see eqs 11 and 12). However, a constant liquid superficial velocity means that the liquid flow rate (*Q*_l_) increases as the length of the absorber increases. In Figure 5, the gas flow rate (*Q*_g_) was equal to 9150.0 L/min, while the liquid flow rate (*Q*_l_) varied from 21.96 to 65.88 L/min. The wet area (*a*_w_) was calculated using eq 11 and the calculated value was 38.6% of the total dry interfacial area (*a*_t_). Figure 5a shows that the average CO_2_ load increased as the absorber length (*L*) increased. The gas phase became increasingly CO_2_ depleted along the horizontal length. The liquid phase falling vertically absorbed less CO_2_ as it interacted with the gas phase moving to the outlet of the equipment. The total CO_2_ load plotted is the average of the different CO_2_ loads calculated at all exit positions. Figure 5b shows that the fraction of CO_2_ absorbed from the gas phase increased as the length of the absorber increased. This behavior was produced by the increased gas phase residence time (L/u_g_).

In Figure 6a,b, we varied the height of the absorber keeping constant the liquid superficial velocity (*u*_l_) and flow rate (*Q*_l_) and the superficial gas velocity (*u*_g_). At constant gas superficial velocity, the gas flow rate (*Q*_g_) increased as the absorber height increased. In Figure 6, the liquid flow rate (*Q*_l_) was equal to 21.96 L/min, while the gas flow rate (*Q*_g_) varied from 2790.8 to 91,500 L/min. The wet area (*a*_w_) was calculated using eq 11, and the calculated value was 38.6% of the total dry interfacial area (a_t_).

Figure 6a shows that the CO_2_ total load increased continuously as the height of the absorber increased. The liquid phase flowing downward interacted with the gas phase with constant concentration in the vertical direction increasing continuously CO_2_ absorption as it reached the bottom of the absorber. Figure 6b shows that the CO_2_ fraction absorbed from the gas phase decreased as the height increased. The gas absorption fraction decreased from top to bottom. This behavior is produced because the increasing CO_2_ concentration in the liquid phase, as it moved downward, decreased the driving force for absorption from the gas phase.

Figure 7a,b depicts the influence of the liquid phase superficial velocity on CO_2_ absorption. In these figures, the gas flow rate (*Q*_g_) was equal to 9150.0 L/min, while the liquid flow rate (*Q*_l_) varied from 21.96 to 65.88 L/min. Figure 7a shows that the CO_2_ total load increased continuously as the liquid superficial velocity increased. The increase in the liquid superficial velocity (*u*_l_) increased the gas–liquid mass transfer area (*a*_w_) from 20 to 45% of the total specific area (*a*_t_). Inspection of Figure 7a shows that the CO_2_ load increase was smaller than the one predicted from the increase in mass transfer area alone. However, at the same time, the increase in liquid superficial velocity decreased the residence time and CO_2_ absorption. We conclude that the decrease in residence time partially offset the increase in CO_2_ absorption produced by the increase in the wet surface area. Figure 7b shows that the CO_2_ fraction absorbed from the gas phase increased as the liquid superficial velocity increased. As time increased, however, the absorption fraction decreased faster for higher liquid superficial velocities until it reached very small values at equilibrium.

Figure 8a,b shows the influence of the gas phase superficial velocity on CO_2_ absorption. In these figures, the liquid flow rate (*Q*_l_) is equal to 21.96 L/min, while the gas flow rate (*Q*_g_) varied from 3660 to 18300 L/min. The wet area (*a*_w_) was calculated using eq 11, and the calculated value was 38.6% of the total dry interfacial area (*a*_t_). Figure 8a shows that the CO_2_ total load increased continuously as the gas superficial velocity increased. The increase in the gas superficial velocity (*u*_g_) increased the molar flow from gas into the liquid phase. Inspection of Figure 8a shows that the CO_2_ load increase was smaller than the one predicted from the increase in mass transfer area alone. The observed CO_2_ uptake values presented in this study varied from 0.333 to 0.656 kg/m^3^h.

It is shown in Figure 8b that the absorption fraction from the gas phase decreased as the superficial gas velocity increased. This behavior was produced by the reduction of the gas phase residence time. We can conclude that the increase in gas flow rate increased the amount absorbed, but at a lower CO_2_ capture efficiency.

The absorption process is a two-step sequential process. The first step involves CO_2_ gas–liquid mass transfer, and the second step involves chemical reaction. Gabitto and Tsouris reported that, at short times, the process is mass transfer controlled while, at longer times, it becomes reaction controlled, as the amino acid concentration significantly decreases.^35^ In this work, we studied the issue by plotting the free CO_2_ liquid phase concentration versus time for typical input values. The results are shown in Figure 9.

**Figure 9:**
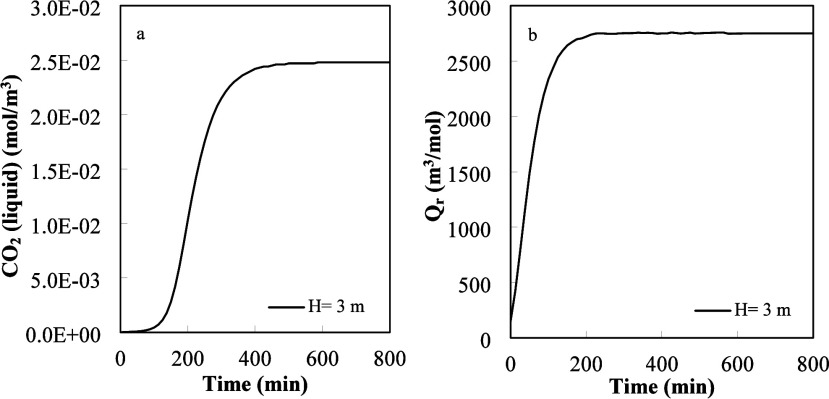
(a) Time variation of free CO_2_ in the liquid phase. (b) Time variation of the eq 1 reaction quotient (*Q*_r_).

The results depicted in Figure 9a are like those reported by Gabitto and Tsouris.^35^ At short times, less than 100 min, the amount of free CO_2_ absorbed was very small showing that the process was mass transfer controlled. After 100 min, the amount of free CO_2_ increased continuously as the process became first mixed controlled and later reaction controlled. At times longer than 400 min, there was very little CO_2_ absorption as the free CO_2_ liquid phase concentration became equal to the equilibrium concentration at that temperature and stopped gas–liquid mass transfer. In Figure 9b, we plotted the value of the calculated reaction quotient (*Q*_r_) for the amino acid CO_2_ absorption, the main absorption reaction (eq 1). These results were calculated using values of the different chemical species under the same operating conditions as in Figure 9a. Figure 9b shows that by times longer than 200 min, the reaction was at equilibrium, i.e., *Q*_r_ = *K*_eq_. Other results, not presented here, showed that all the chemical reactions participating in the reaction scheme (see the Supporting Information) were at equilibrium, or close to equilibrium values, at times longer than 200 min. For longer times, there were some small oscillations produced by changes in individual species concentrations that produced some small extra absorption. For example, eq 5 produced some extra sarcosine salt (SAR^–^); the extra SAR^–^ consumed some CO_2_ dissolved in the liquid phase. The decrease in dissolved CO_2_ produced some CO_2_ absorption from the gas phase, and the cycle repeated. Calculated results show that CO_2_ absorption was very small at longer times.

## 4 Conclusions

CO_2_ absorption in a crossflow absorber has been studied numerically, and a theoretical model predicting the operation of a crossflow absorber was developed by modifying a previously developed countercurrent flow model. The model has been validated by using experimental data for comparison. The calculated results show that the specific CO_2_ load increased with gas and liquid superficial velocities, and equipment height and length. The fraction of CO_2_ absorbed from the gas phase increased with increasing liquid velocity and equipment length but decreased with increasing gas superficial velocity and equipment height. CO_2_ absorption was found to increase with increasing temperature. Carbon dioxide absorption proceeded through a two-step sequential process, mass transfer from gas to liquid, and chemical reaction in an interfacial liquid thin film. An analysis of the rates of the individual steps suggests that, at short times, the absorption process was mass transfer controlled while, at longer times, the process was kinetically controlled. The increase in the amount of CO_2_ dissolved in the liquid phase decreased mass transfer at long times until virtually stopping gas–liquid mass transfer. At medium and longer times, the system reached chemical equilibrium with very small CO_2_ absorption. Based on the results of this work, one can increase CO_2_ absorption by increasing liquid velocity or flow rate and designing a taller and/or wider contactor. The experimentally validated model developed in this work can be combined with a cost model to determine optimal values of contactor dimensions and operating parameters, such as air and liquid flow rates, for a given CO_2_ annual capture rate.

## Supplementary Material



## Notes

Contains reactions for sarcosine CO_2_ absorption, mass balance equations, charge balance equations, kinetic information used in solving the proposed reaction model, variation of average CO_2_ gas phase concentration with absorber length, and output concentration profiles (PDF)

## Notes

The authors did not use AI throughout this project.

## Nomenclature

AA: amino acid
*a*_t_: interface area per unit volume (m^–1^)
*a*_w_: wet interface area per unit volume (m^–1^)
*C*_g_: constant for gas phase mass transfer (dimensionless)
*C*_h_: Packing constant for liquid hold-up (dimensionless)
*C*_l_: constant for liquid phase mass transfer (dimensionless)
*C*_*i*_^*k*^: concentration of *i* species in *k*-phase (mol m^–3^)
*C*_*i*_^g^: species i gas phase concentration (mol m^–3^)
*C*_*i*_^l^: species i liquid phase concentration (mol m^–3^)
*C*_*i*_^l,*^: species *i* liquid phase equilibrium concentration (mol m^–3^)
*C*_*i*,In_^g^: input gas phase concentrations of the *i* component (mol m^–3^)
*C*_*i*,Out_^g^: output gas phase concentrations of *i* species (mol m^–3^)
*C*_pi_^g^: heat capacities of the *i* component (J mol^–1^ K^–1^)
*C*_pi_^l^: heat capacities of the *i* component (J mol^–1^ K^–1^)
*D*: reactor depth (m)
*d*_bs_: gas bubble Sauter diameter (m)
*D*_CO2_: CO_2_ diffusion coefficient (m^2^ s^–1^)
*E*: enhancement factor (dimensionless)
*h*_t_: liquid hold-up per unit volume (dimensionless)
*H*: absorber height (m)
*k*_CO2_^l^: liquid-side CO_2_ mass transfer coefficient (m s^–1^)
*k*_f_: forward specific reaction rate constant (m^3^ mol^–1^ s^–1^)
*k*_r_: reverse specific rate constant (m^3^ mol^–1^ s^–1^)
*K*_*i*_: equilibrium constant for *i* reaction (variable)
*L*: absorber length (m)
*N*_*i*,diff_: molar flow of component i from the gas into the liquid phase (mol m^–3^ s^–1^)
pH: liquid phase pH (dimensionless)
*Q*: heat of reaction (J mol^–1^)
*Q*_g_: gas flow rate (m^3^ s^–1^)
*Q*_l_: liquid flow rate (m^3^ s^–1^)
*R*: alkyl group
*Re*_l_: liquid phase Reynolds number
*R*_gen,*i*_: mol of species i generated by chemical reaction (mol m^–3^ s^–1^)
*T* ^g^: temperature of the gas phase (K)
*T*_o_^g^: temperature of the input gas stream (K)
*T* ^l^: temperature of the liquid stream (K)
*U*_T_: global heat transfer coefficient (J m^–2^ K^–1^ s^–1^)
*u*_g_: gas superficial velocity (m s^–1^)
*u*_l_: liquid superficial velocity (m s^–1^)
*V*_l_: liquid phase volume (m^3^)
*V*_T_: total reactor volume (m^3^)
*W*: absorber width (m)
**Greek letters**
Δ*H*_vap,*i*_: heat of vaporization for the *i* species (J mol^–1^)
Δ*H*_R_: heat released by chemical reaction (J mol^–1^)
ε: bed void fraction (dimensionless)
ε_g_: gas phase hold-up (*V*_g_/*V*_T_)
μ_*i*_: dynamic viscosity of the *i* phase (Pa s)
ρ_l_: liquid phase density (kg m^–3^)
σ_l_: liquid phase surface tension (N m^–1^)
