# Supplementary material for: Direct Air Capture Using Aqueous Amino Acid Solvents in a Crossflow Absorber
Source: Ind Eng Chem Res. 2025 Dec 22;65(1):714–23. doi: 10.1021/acs.iecr.5c02551 (PMC12810392; doi:10.1021/acs.iecr.5c02551)
Supplement: Supplementary file 1 [file ie5c02551_si_001.pdf]

Supporting Information

Direct Air Capture using Aqueous Amino-Acid Solvents in a Crossflow Absorber

Jorge Gabitto,[1]* Abishek Kasturi,[2] Gyoung Gug Jang,[2]
Radu Custelcean,[2] Costas Tsouris[2]

[1] Chemical Engineering Department, Prairie View A&M University

[2] Oak Ridge National Laboratory, P.O. Box 2008, Oak Ridge, TN, 37831

* Corresponding Author: jfgabitto@pvamu.edu

Reactions for Sarcosine CO_2 Absorption

$CO_2\ (aq.) + 2\ SAR^- \overset{K_1}{\Leftrightarrow} SAR^-(CO_2H) + SARH$ (I)

$CO_2\ (aq.) + H_2O \overset{K_1}{\Leftrightarrow} HCO_3{}^- + H^+\ (aq.)$ (II)

$CO_2\ (aq.) + OH^- \overset{K_2}{\Leftrightarrow} HCO_3{}^-\ (aq.)$ (III)

$H_2O \overset{K_4}{\Leftrightarrow} H^+ + OH^-$ (IV)

$SARH \overset{K_5}{\Leftrightarrow} SAR^- + H^+$ (V)

$SAR^-(CO_2^-) + H_2O \overset{K_6}{\Leftrightarrow} HCO_3{}^-\ (aq.) + SAR^-$ (VI)

$HCO_3{}^-(aq.) + OH^- \overset{K_7}{\Leftrightarrow} CO_3{}^{2-}$ (VII)

Mass Balance Equations

$[SAR^-]_o = [SAR^-] + [SARH] + [SAR^-CO_2H)] + [SAR^-(CO_2^-)]$ (VIII)

$CO_2Load = [HCO_3{}^-] + [CO_3{}^{2-}] + [\ CO_2(liq.)] + [SAR^-(CO_2^-)]$ (IX)

Charge Balance Equations

$$[K^+] + [H^+] = [SAR^-] + [OH^-] + 2\,[SAR^-(CO_2^-)] + [HCO_3{}^-] + 2\,[CO_3{}^{2-}] \quad \text{(X)}$$

Table S1. Kinetic information used in solving the proposed reaction model

Reaction Number	Forward Rate (k_{fi})	Equilibrium Constant (K_i)	Reverse Rate (k_{ri})
I	$k_{1f} = 3.91 \times 10^{10}$ exp(-6863/T_l)	K_1 = 1.24E-9 × exp(1571.24./T_l)	$k_{1r} = k_{1f} / K_1$
II	k_{2f} = 0.024 (s^{-1})	$K_2 = 1 \times 10^6$ exp[231.47 – (12092.1/T_l)] – 6.78 Ln(T_l)	$k_{2r} = k_{2f} / K_2$
III	$k_{3f} = 10^{-3} \times$ exp[(3.65 – 2.9/T_l)] ($mM^{-1}s^{-1}$)	$K_3 = K_2 / K_4$	$k_{3r} = k_{3f} / K_3$
IV	k_{4f} = 0.024 (s^{-1})	$K_4 = 1 \times 10^6 \times$ exp[(-7.09 × exp(203.33/T_l)]	$k_{4r} = k_{4f} / K_4$
V	$k_{5f} = 1 \times 10^{-5}$ (s^{-1})	$K_5 = 2.93 \times 10^3 \times$ exp[-7387.8/T_l)]	$k_{5r} = k_{5f} / K_5$
VI	k_{6f} = 0.002 (s^{-1})	$K_6 = 1 \times 10^6$ exp[1.28 –(8456.2/T_l)]	$k_{6r} = k_{6f} / K_6$
VII	k_{7f} = 600 (s^{-1})	K_7 = exp [(1568.9/T_l) – 2.58 – 6.74 × $10^{-3} \times T_l$)]	$k_{7r} = k_{7f} / K_7$

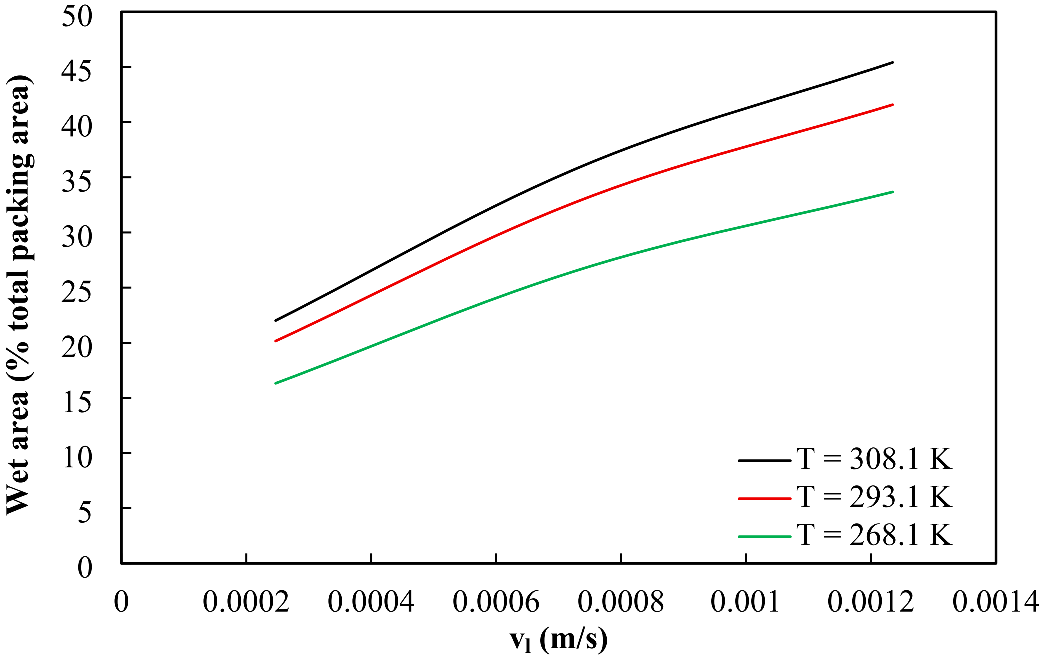

Figure S1. Variation of percentage of wet area (100 x a_w/a_t) with superficial velocity (v_l) and temperature.

Variation of Average CO_2 Gas Phase Concentration with Absorber Length

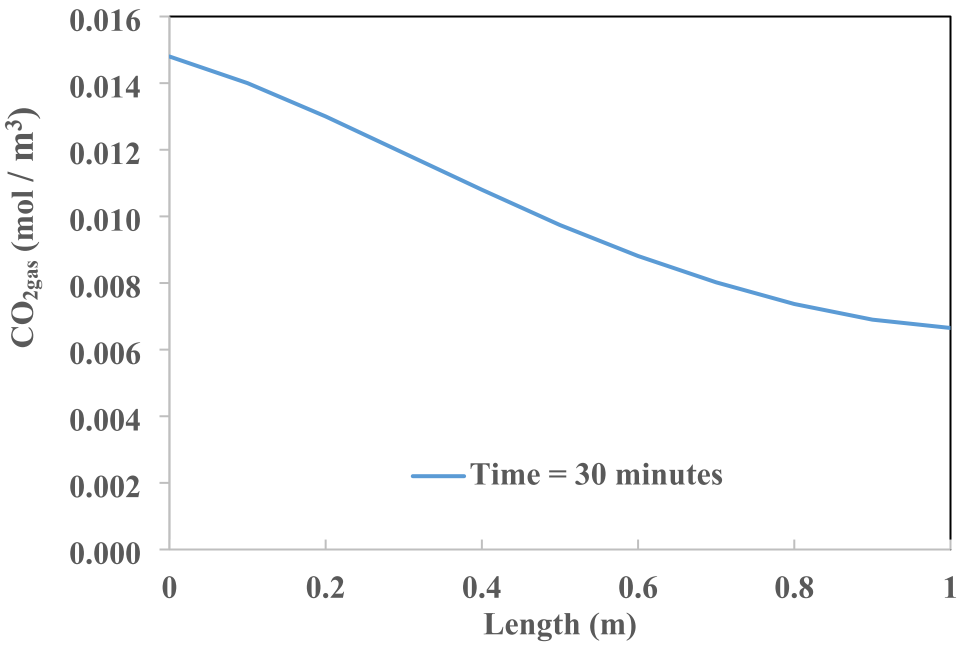

Figure S2. The vertically averaged CO_2 concentration profile in the gas phase along the absorber length. Data: u_l= 7.4E-4 m/s, u_g = 0.5 m/s, H = 0.24 m, W = 0.3 m, L = 1 m, T = 295.1 K, sarcosine concentration = 1 M, and input CO_2 concentration= 0.0184 mol/m^3.

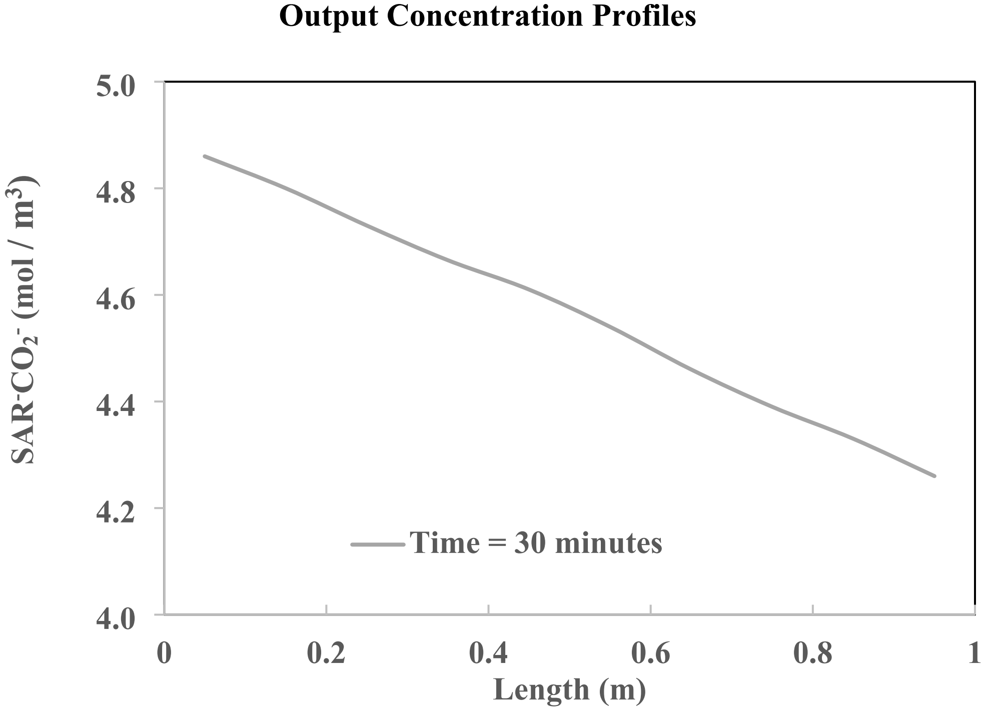

Figure S3. Variation of carbamate ($SAR^-CO_2^-$) output concentration with absorber length. Data: u_l = 7.4E-4 m/s, u_g = 0.5 m/s, H = 0.24 m, W = 0.3 m, L = 1 m, T = 295.1 K, sarcosine concentration = 1 M, and input CO_2 molar fraction = 3.28E-4. At short times CO_2 is stored as sarcosine carbamate ($SAR^-CO_2^-$). The concentration of the sarcosine carbamate decreases continuously from the air input (L = 0 m) to the air output (L = 1 m). The alkaline sarcosine (SAR^-) concentration, mostly constant at short times interacts with a decreasing gas phase CO_2 concentration, see Figure S2.

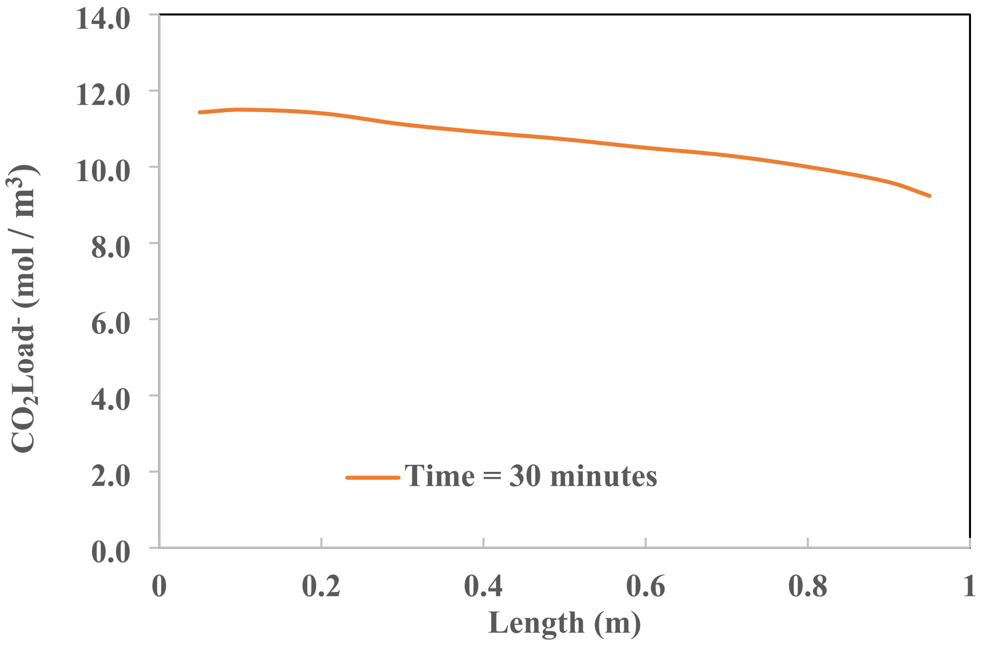

Figure S4. Variation of CO_2Load ($SAR^-CO_2^-$ + HCO_3^- +CO_3^{2-}) output concentration with absorber length. Data: u_l = 7.4E-4 m/s, u_g = 0.5 m/s, H = 0.24 m, W = 0.3 m, L = 1 m, T = 295.1 K, sarcosine concentration = 1 M, and input CO_2 molar fraction = 3.28E-4.

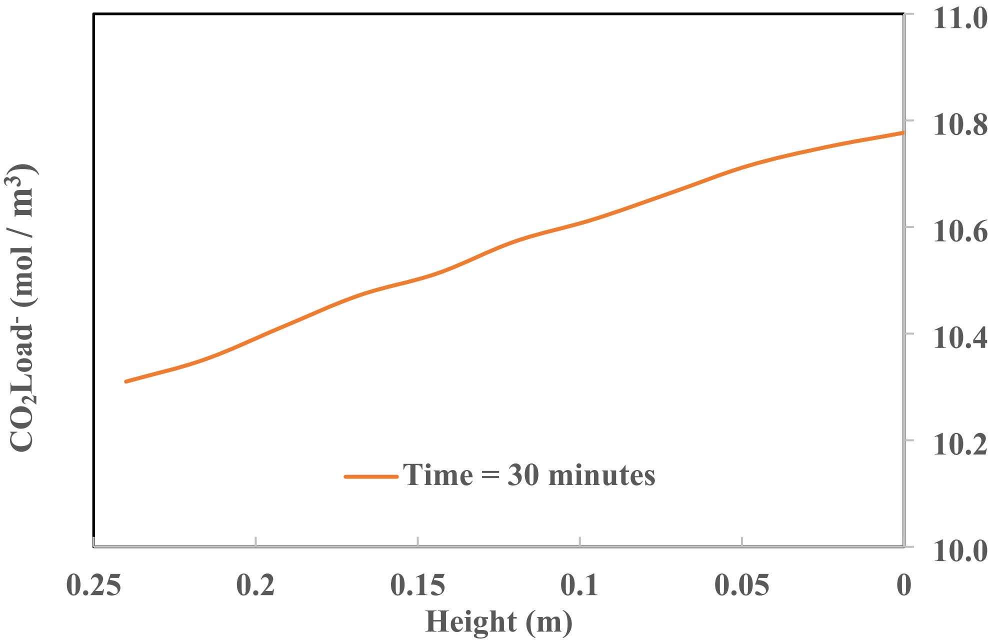

Figure S5. Variation of CO_2Load ($SAR^-CO_2^-$ + HCO_3^- +CO_3^{2-}) output concentration with absorber height. Data: u_l = 7.4E-4 m/s, u_g = 0.5 m/s, H = 0.24 m, W = 0.3 m, L = 1 m, T = 295.1 K, sarcosine concentration = 1 M, and input CO_2 molar fraction = 3.28E-4. Every point is an average of a horizontal profile. The solvent enters at H = 0.24 m and exits at H= 0 m.
